# Experimental Band Structure of Pb(Zr,Ti)O_3_: Mechanism of Ferroelectric Stabilization

**DOI:** 10.1002/advs.202205476

**Published:** 2023-01-02

**Authors:** Dana Georgeta Popescu, Marius Adrian Husanu, Procopios Christou Constantinou, Lucian Dragos Filip, Lucian Trupina, Cristina Ioana Bucur, Iuliana Pasuk, Cristina Chirila, Luminita Mirela Hrib, Viorica Stancu, Lucian Pintilie, Thorsten Schmitt, Cristian Mihail Teodorescu, Vladimir N. Strocov

**Affiliations:** ^1^ National Institute of Materials Physics Atomistilor 405A Magurele 077125 Romania; ^2^ Swiss Light Source Paul Scherrer Institute Villigen‐PSI 5232 Switzerland

**Keywords:** buried interfaces, ferroelectric‐dependent band structure, ferroelectrics, soft X‐ray angle resolved photoelectron spectroscopy

## Abstract

Pb(Zr,Ti)O_3_ (PZT) is the most common ferroelectric (FE) material widely used in solid‐state technology. Despite intense studies of PZT over decades, its intrinsic band structure, electron energy depending on 3D momentum k, is still unknown. Here, Pb(Zr_0.2_Ti_0.8_)O_3_ using soft‐X‐ray angle‐resolved photoelectron spectroscopy (ARPES) is explored. The enhanced photoelectron escape depth in this photon energy range allows sharp intrinsic definition of the out‐of‐plane momentum k and thereby of the full 3D band structure. Furthermore, the problem of sample charging due to the inherently insulating nature of PZT is solved by using thin‐film PZT samples, where a thickness‐induced self‐doping results in their heavy doping. For the first time, the soft‐X‐ray ARPES experiments deliver the intrinsic 3D band structure of PZT as well as the FE‐polarization dependent electrostatic potential profile across the PZT film deposited on SrTiO_3_ and La*_x_* SrMn_1−_ *_x_* O_3_ substrates. The negative charges near the surface, required to stabilize the FE state pointing away from the sample (P+), are identified as oxygen vacancies creating localized in‐gap states below the Fermi energy. For the opposite polarization state (P−), the positive charges near the surface are identified as cation vacancies resulting from non‐ideal stoichiometry of the PZT film as deduced from quantitative XPS measurements.

## Graphical abstract

The P+ ferroelectric state, pointing away from the sample requires negative charge accumulation close to the surface (a) while positive charges accumulate close to the surface of P− samples (b). For P+ samples, such off‐stoichiometry reveals in‐gap, oxygen vacancies‐related band (c), absent for P− samples (d). X‐ray photoelectron spectroscopy measurements reveal Ti/Zr vacancies at the P− surface.

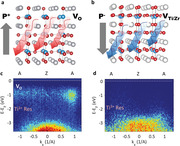

## 1 Introduction

Every ferroic order involves the breaking of a symmetry operation: ferromagnetism (FM) breaks time inversion, ferroelasticity (FS) breaks the spatial rotation symmetry, and ferroelectricity (FE) breaks the inversion symmetry.^[^ ^1^ ^]^

In condensed matter, the macroscopic fingerprint of ferroelectricity is the spontaneous electric polarization P, stable in time and reversible under external electric fields exceeding the coercive field. It is well established that the paraelectric to ferroelectric transition involves a structural transition from high to low symmetry, with consequent displacement and off‐centering of the atoms from their symmetric positions.^[^ ^2^, ^3^, ^4^ ^]^ At microscopic level, such non‐centrosymmetric configuration of the atomic positions in the unit cell is consistent with a picture of dipoles aligned along the direction of the internal electric field.

Current in‐use applications of FE materials range from non‐volatile memories,^[^ ^5^ ^]^ sensors,^[^ ^6^, ^7^ ^]^ transducers^[^ ^8^, ^9^ ^]^ to catalysis^[^ ^10^, ^11^ ^]^ and photovoltaics.^[^ ^12^, ^13^ ^]^ For solar energy storage and conversion,^[^ ^14^, ^15^ ^]^ the strong internal field of FEs is an essential ingredient to efficiently separate the electrons from holes.^[^ ^13^, ^16^ ^]^ A major drawback is their generally wide band‐gap which drastically limits the absorption efficiency in the visible range. Strategies to circumvent this shortcoming include heterostructuring,^[^ ^17^ ^]^ doping^[^ ^18^ ^]^ and/or defect engineering^[^ ^19^ ^]^ in order to either decrease the electronic band gap or to introduce localized electronic states in the band‐gap, which may account for light absorption at convenient energies in the visible range. Assessing the energy and width of these localized levels or possible hybridization mechanisms between the dopant and the bulk band structure are critical to understanding and tuning the properties of the ferro‐functional systems.

The best known FE material is PbZr*_x_* Ti_1−_ *_x_* O_3_ (PZT), which in its aristotype, or fundamental unit cell, is described by a tetragonal (TG) ABO_3_ formula, having Pb in the corners and Ti/Zr in the center of the O_6_ octahedra. It is accepted that this is only a general description and lower symmetries such as orthorhombic, rhombohedral (RH) or monoclinic, coexist with the TG phase,^[^ ^20^ ^]^ with their variable coexistence depending on the Zr amount or temperature. Tuning these parameters is a practical route to increase piezoelectricity^[^ ^21^ ^]^ or to control the domain wall patterns.^[^ ^22^ ^]^ At the *x* = 0.2 value of Zr content, the bulk phase has the TG symmetry with the permanent dipole moment and stable FE polarization generated by displacement along the *c* axis of the cations with respect to the oxygen atoms.^[^ ^5^, ^23^ ^]^ In thin films, the accompanying electrostatic effects associated to such dipole orientation is the accumulation of opposed charges at its extremities, defining a depolarizing field (DF) which opposes the internal field and tends to cancel the FE polarization.

For any application based on FEs, it is important to consider the mechanisms (intrinsic and extrinsic) which contribute to the stabilization of a well‐defined FE state and to isolate their impact on the electronic properties.

Possible extrinsic compensation mechanisms include screening of the DF by the carriers of a metallic electrode; or, adsorption of polar molecules at the surface of the FE. The resulting consequence is the modulation of the charge density close to the contact region, in the outer material.

Intrinsic mechanisms involve migration across the FE material^[^ ^24^, ^25^ ^]^ of the already existing positive (holes, ionized donor impurities, or *p*‐type dopants) and negative charges (electrons, ionized acceptor impurities, or *n*‐type dopants), intentionally introduced^[^ ^26^ ^]^ or resulting from self‐doping.^[^ ^27^ ^]^ Such charge reorganization is accompanied by band bending which manifests either in the outer material for purely extrinsic compensation or within the FE in the case of intrinsic mechanisms. For imperfect screening of the DF by the metallic electrodes, the compensation mechanism is a combination of intrinsic and extrinsic contributions, accompanied by band bending in opposite directions in both the FE and the outer material (metallic electrode or contamination layer).^[^ ^25^ ^]^ The FE‐induced potential profile adds at the material‐dependent band alignment (derived from joining systems with different work functions, *W*_f_). This impacts the hopping of charges across the interface. It also explains the preferential stabilization of opposite FE states of a layer when grown on substrates with different *W*_f_s and different conducting character such as metallic, insulator, *n* or *p*‐doped.

The most direct method to access the intrinsic electronic structure of materials encoded in its band structure is angle‐resolved photoelectron spectroscopy (ARPES). Tracking the experimental band structure of insulating materials, FE ones in particular, is challenging due to charging effects and broadening of the spectra due to the band‐bending in the region close to the surface. With some notable exceptions,^[^ ^28^, ^29^, ^30^ ^]^ the FEs remain underrepresented in ARPES measurements compared with their FM ferroic analogues. GeTe and GeMnTe were studied extensively,^[^ ^29^, ^30^ ^]^ however, unlike FE oxides which are insulators, GeTe‐derived compounds are semiconductors with only small band‐gap and large, p‐type conductivity. The only k‐resolved study of a FE oxide remains up to now only BaTiO_3_, with its band structure recorded in surface‐sensitive measurements, in the heavily *n*‐doped case.^[^ ^28^ ^]^

Soft X‐ray (SX)‐ARPES extends the probing region more toward the bulk, away from the surface band‐bending potential of ferroelectrics, while increasing the *k*_z_ resolution, critical for 3D materials such as Pb(Zr,Ti)O_3_ and BaTiO_3_. Moreover, SX‐ARPES is naturally suited to selectively extract the fingerprints of possible hybridization between the impurity states and the bulk band structure using resonant photoemission,^[^ ^31^, ^32^, ^33^ ^]^ specifically shining out the contribution of impurity states to the total ARPES signal.

Here, we use SX‐ARPES to explore the 3D band structure of PZT with Zr doping *x* = 0.2, prepared in opposite FE states when grown on substrates with different *W*_f_ and *n*/*p* conduction character, focusing on the spectral signatures of the charge carriers stabilizing its polarization state. By separating the FE‐induced effects from the substrate‐induced ones, we clarify fundamental aspects related to the electronic structure of oxide interfaces and formulate further directions to enrich the functionality of multiferroic systems.

The structure of the paper is the following:

I. The first section explores the k‐resolved valence band structure of PZT in two opposed FE states and separates the contribution of the substrate‐induced distortions from the FE‐induced one.
II. The second section identifies the FE polarization‐dependent band alignment mechanism of PZT in two opposed FE states and clarifies the mechanism of FE compensation from combined band structure and core levels analysis.
III. The third section addresses the effects of X‐ray irradiation and identifies the distinct mechanism of charged vacancies creation under external perturbations in order to stabilize and preserve the FE state.
IV. The last section is devoted to outlook and conclusions.

## 2 Results and Discussion

### 2.1 k‐Resolved Valence Band Structure

Two PZT samples with thickness of 5 nm were grown by pulsed laser deposition on LaSrMnO_3_ (LSMO) buffered TiO_2_‐terminated (001) SrTiO_3_ (STO) substrate and on TiO_2_‐terminated (001), Nb‐doped SrTiO_3_ (Nb:STO). Throughout the paper we will refer to them as DW respectively UP samples. Detailed explanations on the growth protocol are given in the Experimental Section.

Such a small thickness means that: i) the FE state is an out‐of‐plane, mostly single domain, with P oriented either inward, toward the substrate (P−) or outward, toward the vacuum (P+); and ii) the screening of the DF and the stabilization of the FE order is maintained by a significant concentration of charge carriers existing in the thin PZT layer (charged impurities, cation vacancies [CV]).^[^ ^27^, ^34^ ^]^ These carriers also protect from the charging effects, allowing direct observation of the k‐resolved band structure.

We select the two different substrates due to their larger, respectively smaller work‐functions (*W*_f_): 𝛟_LSMO_ = 4.9–5.1 eV,^[^ ^35^, ^36^ ^]^ 𝛟_Nb:STO_ = 4.1–4.2 eV^[^ ^36^ ^]^ compared to PZT, 𝛟_PZT_ = 4.5 eV.^[^ ^34^ ^]^ The *W*_f_‐induced band lineup at the interface with the substrate define a material‐dependent band bending, Δ𝛟^DW(UP)^ which drive the migration of negative (positive) charges from PZT at the bottom interface. In combination with the available positive (in LSMO) and negative (in Nb:STO) charges in the substrate, we expected the stabilization of well‐defined, opposed out‐of‐plane P−(P+) FE states of PZT.

XRD measurements performed at the room temperature (Figure S1, Supporting Information) on both samples indicate that PZT is fully strained at the in‐plane lattice constant of the STO substrate, and elongated along the *c* axis. The ratio between the out‐of‐plane (*c*) and the in‐plane (*a*) lattice parameters of the pseudo‐cubic unit cell, *c/a* ≈ 1.07–1.08 exceeds the *c/a* ≈ 1.04–1.05 value of fully relaxed, bulk PZT.^[^ ^27^ ^]^ Such geometry involves strong cation displacement from the energetically unfavorable centrosymmetric position,^[^ ^37^, ^38^ ^]^ indirectly supporting the ferroelectric character of the thin films. We identify the distinct out‐of‐plane FE polarization, pointing outward (P+) or inward (P−) in local piezoresponse force microscopy measurements (Figure S2, Supporting Information) performed on the two samples after the synchrotron measurements. The result identifies PZT in the UP sample with FE state oriented away from the surface (P+) while PZT in DW, features FE polarization oriented inward (P−).

We will now explore the distinct hallmarks of the different crystalline structure of the substrates: cubic Nb:STO (UP sample) and RH‐distorted LSMO^[^ ^39^ ^]^ (DW sample), emphasizing the signature of the different crystalline state and FE polarization in the electronic structure of PZT.

**Figure 1:**
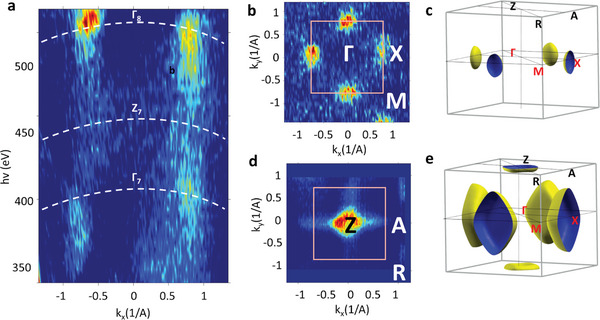
Out‐of‐plane iso‐energy (iso‐E) maps of PZT recorded by varying *hv* between 350 and 520 eV while keeping the k_||_ in the XΓZ plane of the bulk Brillouin zone (a). (*k_x_* − *k_y_*) iso‐E in the ΓMX plane at the VB maximum—VBM (b) and in the ZAR plane at 0.5 eV below the VBM (d). The calculated iso‐E surface at VBM (c) and 0.5 eV below VMB (e).

We investigate first the k‐space topology of PZT in the UP sample with tetragonal unit cell (u.c.), by navigating along the out‐of‐plane *k*_z_ direction. In **Figure** **1**a, the photon energy ℎ*ν* is varied between 350 and 520 eV at constant *k*_||_. The resulting iso‐energy (iso‐*E*) map in the XΓZ plane of the bulk Brillouin zone (BZ), presented in Figure 1a, identifies the valence band maximum (VBM) in the X point at a binding energy (BE) of 2.2 eV relative to the Fermi level.

The (*k_x_* − *k*_y_) iso‐*E* map recorded on UP sample in the XΓM plane at the VBM using *hv* = 520 eV (Figure 1b) derives from the square‐like symmetry of the PZT in the *ab* plane in accordance with the iso‐*E* surface derived from DFT calculations for the tetragonal PZT u.c. (Figure 1c).

The iso‐*E* map recorded in the ZAR plane 0.5 eV below VBM with *hv* = 465 eV (Figure 1d) also shows the DFT‐predicted feature centered in the Z point for the tetragonal u.c. (Figure 1e).

Such topology of the k‐space is particular to this heavily strained PZT layer, whereas in fully relaxed, bulk PZT with the *c*/*a* ratio of 1.05, the features in the ZRA plane open in the R point, not in the Z point as seen in Figure S3, Supporting Information.

**Figure 2:**
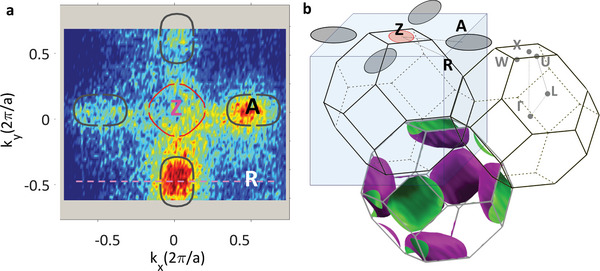
Signature of RH reconstruction. In‐plane (*k_x_*,*k_y_*) iso‐E maps of PZT recorded with *hv* = 465 eV at 0.5 eV below the VBM in the ZAR plane of the TG cell. The red line indicates the calculated isocontour at the corresponding energy assuming TG geometry of the unit cell. Gray contours represent the additional signature of the RH‐distorted unit cell for DW (a). Such a signature is absent for the UP sample. The signature of the RH distortion is rendered into the TG unit cell in (b), with the iso‐energy surfaces calculated at the same energy, 0.5 eV below VBM, as the experimental maps.

However, for the DW sample, the iso‐*E* map recorded in the ZAR plane 0.5 eV below VB is not consistent with the iso‐*E* calculated in the tetragonal cell as shown in **Figure** **2**a. It shows in addition to the expected signature of the hole‐like band in the Z point, four elliptical‐shaped features centered in the A points of the k‐space. Their appearance is consistent with a RH reconstruction of the PZT unit cell. This is evident from the calculated 3D iso‐*E* surface of PZT in a RH unit cell at the same energy with respect to the VBM as in the experimental (*k_x_*,*k*_y_) map and presented in Figure 2b. The calculated iso‐*E* map shows that the major axis of the pocket around the *A* point derives from the projection of the *WXW* direction of the RH cell on the (001) direction of the TG cell. In turn, the minor axis of the A‐centered ellipse results from projecting the *UXU* direction of the RH cell on the (001) plane of the TG‐cell BZ.

Indeed, it has been shown recently how across the interface between transition metal oxides,^[^ ^40^ ^]^ the magnitude and pattern of the octahedral tilts extend the picture of epitaxial‐induced strain by the substrate,^[^ ^41^ ^]^ to additional structural reconstruction propagating from the substrate into the top layer across a thickness of 2–3 nm. Such an effect explains the morphing of the substrate crystalline structure into PZT top layer The mechanism of imprinting the substrate crystal structure into the top epitaxial layer is independent of the ferroelectric state, involving a combination of substrate‐induced strain and continuity of the octahedral rotations.^[^ ^42^ ^]^

Notably, the reconstruction observed for PZT when grown on LSMO keeps up to the room temperature, excluding the scenario of a low‐temperature RH reconstruction, characteristic to many perovskite oxides in the bulk phase^[^ ^43^ ^]^ (Figure S4, Supporting Information), with the additional temperature‐dependent X‐ray absorption measurements further supporting the different crystalline state of PZT grown on the two different substrates (Figure S5, Supporting Information, and accompanying discussion).

The effect of the RH distortion which manifests as folding of the fundamental pseudocubic unit cell along the k‐space diagonal is illustrated for the band structure recorded across the RAR and XMX direction in Figure S6, Supporting Information, and the mechanism described in the accompanying discussion.

**Figure 3:**
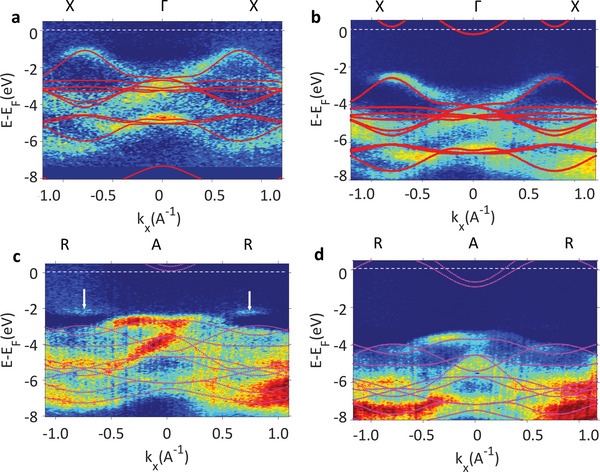
Experimental band structure recorded along XΓX (a,b) and RAR (c,d) directions of PZT grown on LSMO (DW) (a,c) and Nb:STO (UP) (b,d) samples. The calculated band structures along the corresponding directions are overlaid with red lines showing the DFT calculations for the bulk PZT in the tetragonal unit cell. The arrows in (c) indicate the signature of rhombohedrally distorted PZT on LSMO substrate.

The electronic band structure recorded along two high symmetry directions, XΓX and RAR of the tetragonal BZ is given in **Figure** **3**. The bands in the XΓX directions derive from Pb 4*s* states while those along RAR correspond to O 2*p* states hybridized with Ti 2*p* as seen in Figure S7, Supporting Information. PZT band dispersions in UP and DW samples are qualitatively similar, however, rigidly shifted by 1.25 eV one with respect to the other. This indicates an existing offset *Λ*, of the surface potential which results from the FE‐induced band bending. Hence, the electronic states of PZT in the UP sample are displaced toward higher BE compared to PZT in DW. Such variation of the potential inside PZT is the standard indication of the opposite, out of plane FE states^[^ ^25^, ^44^, ^45^, ^46^, ^47^, ^48^, ^49^ ^]^ in free‐standing ferroelectrics. It occurs in principle when negative/positive charge carriers accumulate close to the surfaces in order to screen the accompanying DF established in the material with opposed P+/P− FE states.^[^ ^50^, ^51^ ^]^

The sharp band structure obtained in our ARPES measurements suggests that the expected band bending close to the surface is small, not exceeding 200–300 meV,^[^ ^52^, ^53^, ^54^, ^55^ ^]^ as otherwise it would smear the entire band structure and obscure the observation of clearly dispersing electronic states. This is not surprising since the potential inside the FE may flatten in the proximity of the interface with metallic electrodes or polar adsorbates. Such effect is accompanied by bending of the potential inside the electrode or in the contamination layer in the opposite direction from that of the FE due to negative/positive charge accumulation in the outer material,^[^ ^25^, ^48^, ^49^ ^]^ at the interface with P+/P− FEs.

Most important, the well‐defined electronic dispersions observed in our ARPES experiments performed on samples which are normally strongly insulating indicates the existence of a considerable amount of free carriers^[^ ^34^ ^]^ which prevents the inherent charging effects in photoemission. Their origin was discussed before and the mechanism identified in the self‐doping effect which develops as a route to stabilize the FE state in thin films.^[^ ^27^ ^]^ However, their explicit signature in the band structure has not been yet identified.

We will now clarify the mechanism of band alignment from the analysis of the core‐levels recorded on both UP and DW samples, identifying the contamination layer as a source of imperfect screening for the DF at the PZT surface. Consequently, the remaining uncompensated field, by opposing the FE‐induced field, tends to reduce the band bending potential close to the surface.^[^ ^25^ ^]^

### 2.2 Polarization‐Dependent Band Alignment Mechanism

Assuming that we have only the un‐screened, fixed polarization charges at the top and bottom extremities of a FE, the internal field should be described by a linear variation of the potential, rigidly followed by the entire electronic structure, including both the valence states and the deep core levels. In order to overcome this energetically unfavorable situation and compensate for the depolarizing field (DF), thick films will develop domains with different FE polarization and minimize the total energy^[^ ^16^, ^27^ ^]^ while in thin films, in the absence of compensation charges, the ferroelectricity may be simply suppressed.^[^ ^56^ ^]^

In order to sustain their FE state, thin films compensate for the DF through intrinsic or extrinsic mechanisms. Intrinsic one involves migration of charge carriers already available in the film, resulting in creation of negative and positive charge sheets at the opposite surfaces of the layer. These charge carriers are created through spontaneous alteration of the ideal stoichiometry during the growth with creation of cation or oxygen vacancies.^[^ ^27^ ^]^

Extrinsic one involves opposite charge accumulation or depletion with respect to the fixed polarization charges from the FE material, in the outer material: the metallic electrode or inside the contamination layer at the surface with the air in order to screen the DF. Such charge modulation effect induced into the joining material exceeds a pure electrostatic picture as the electric field outside the ferroelectric is rigorously zero, and should not impact the electronic structure of the joining material. Nevertheless, it depends on two factors: i) on the particular band alignment at the metal/FE or FE/contamination layer interfaces which involves both the work function difference between the FE and the metallic contact or surface contamination layer and the FE state.^[^ ^25^, ^34^ ^]^ The band alignment picture and the resulting potential profile across the FE defines the regions with positive/negative charge accumulation at the extremities of the FE. Under applied bias, it controls how and if the required electrons and/or holes are injected across the interface and how, by diffusing through material, accumulate at the surface/interfaces of the FE^[^ ^16^, ^25^, ^46^, ^47^, ^57^ ^]^ in order to compensate for the DF. This further translates into material‐dependent potential inside the FE as well as in the joining contacts; and ii) on the polar character of the interface where the electric dipoles possibly resulting at the interface may locally account for additional charges required to stabilize the FE state. Importantly, the dipolar field defined by the fixed polarization charges in the FE and the mobile depolarizing charges localized at the surface or interface decays as *d* ^−3^, where *d* is the distance from the surface dipole, confining the induced charge modulation at the first unit cell of the metallic contact.^[^ ^58^ ^]^

**Figure 4:**
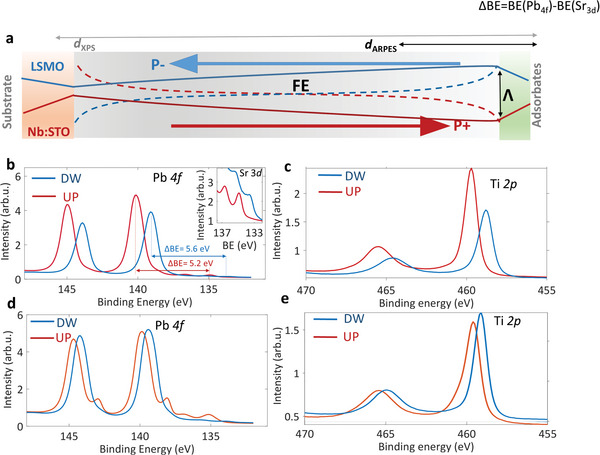
Band bending mechanism from the core levels. Band bending at the PZT surface as a function of the ferroelectric state. a) Sketch of the band bending potential along the UP and DW samples. b,c) Pb 4f and Ti 2p core level spectra of DW (blue) and UP (red)— samples recorded at 12 K and after 20 min of continuous exposure to the X‐ray beam. The same spectra recorded at room temperature are presented in (d,e).

The mechanism of band alignment at the interface of the PZT samples with the substrate and at the surface depending on their FE state is given in the sketch from **Figure** **4**a. It shows the variation of the potential inside the FE film going from the surface toward the interface with the substrate and is deduced by correlating the surface sensitive ARPES data with more bulk sensitive results of the core levels, which extend the probing region down to the interface with the substrate. The dashed line represents the potential profile when opposite charges accumulate into the FE film close to the interfaces for screening the DF. With full lines are qualitatively traced the potentials *V*(*z*) inside our FE films, for both UP and DW samples, derived from the experimental data. The relative BE shift of 1.25 eV between the UP and DW samples identified in the ARPES measurements and the sharp band dispersions is compatible with the small band bending close to the surface and with the observed FE‐induced band offset over the first ≈ 1–2 nm close to the surface.

Going toward the bottom interface, we extract the information on the band lineup by analyzing the BE difference between the UP and DW samples of the Pb 4*f*_7/2_ core levels, Δ_BE_(Pb) = Pb 4*f*_7/2_^(UP)^‐Pb 4*f*_7/2_^(DW)^ (Figure 3b) and Ti 2*p*_3/2_, Δ_BE_(Ti) = Ti 2*p*_3/2_^(UP)^‐Ti 2*p*_3/2_^(DW)^ (Figure 3c). The calculated inelastic mean free paths (𝝀) of Pb 4*f* and Ti 2*p* photoelectrons corresponding to our excitation energy of 1100 eV are in the 2.5 nm‐3 nm range.^[^ ^59^ ^]^ This translates in a probing depth *l* ≈ 3 *λ*, enough to penetrate through the thin FE layer and record the Sr 3*d* signal from the substrate as well. At the bottom interface, the band bending between PZT and the LSMO|Nb:STO substrates in the DW/UP samples and the corresponding V(*z*) profile follows the expected trend derived from their *W*_f_ differences. More exactly, with the values for *W*_f_: 𝛟_PZT_ = 4.5 eV,^[^ ^34^ ^]^ 𝛟_Nb:STO_ = 4.1–4.2 eV^[^ ^36^ ^]^ and 𝛟_LSMO_ = 4.9–5.1 eV,^[^ ^35^, ^36^ ^]^ we estimate Δ𝛟^UP^ = 𝛟_PZT_‐𝛟_Nb:STO_ = 0.4 eV, corresponding to upward band bending at the bottom interface and consequent shift toward lower BEs across the bulk of PZT in the UP sample. For DW sample, Δ𝛟^DW^ = 𝛟_PZT_‐𝛟_LSMO_ = ‐0.3 eV identifies the downward band bending of PZT at the LSMO substrate, accompanied by a shift toward higher BEs of the electronic structure in the FE layer close to the PZT|LSMO interface.

In addition, the large Δ_BE_(Sr) = Sr 3*d*_5/2_^(UP)^ − Sr 3*d*_5/2_^(DW)^ = 1.1 eV in the Nb:STO and LSMO substrate (Figure 3b, inset, and **Table** **1**), by exceeding Δ_BE_(Pb) = 1.0 eV and Δ_BE_(Ti) = 0.9 eV indicates the opposite variation of V(*z*) in the two substrates of UP/DW samples. In the UP sample, it decreases and consequently Sr 3*d* increases to higher BEs, with the opposite increase of V(*z*) in DW and the shift toward lower BEs of its electronic structure.

**Table 1:** Binding energies. Binding energies of Ti 2*p* ^3/2^, Pb 4*f* ^7/2^ core levels from the FE layer and Sr 3*d* ^5/2^ from the substrate in the DW and UP samples, recorded at *T* = 12 K and at room temperature

	RT						*T* = 12 K		
	Freshly exposed			Saturated			‐		
Core level	Ti 2*p*	Pb 4*f*	Sr 3*d*	Ti 2*p*	Pb 4*f*	Sr 3*d*	Ti 2*p*	Pb 4*f*	Sr 3*d*
BE ^(DW)^ [eV]	459.1	139.4	133.8	459.0	139.4	133.8	458.8	139.1	133.5
BE ^(UP)^ [eV]	459.8	140.0	134.9	459.6	139.8	135.1	459.7	140.1	134.9

Such opposite variation is not expected for the *W*_f_‐dependent band alignment at metal|FE interfaces where the metal band structure and its V(*z*) should in principle remain immune to the interface formation. This rather points at a FE‐induced alteration of the carrier density into LSMO and Nb:STO substrates as well, where close to the interface with PZT, the substrate supplies positive/negative carriers to screen the fixed interface dipole charges^[^ ^24^, ^58^ ^]^ and stabilize the UP/DW FE state. Accordingly, V(*z*) in Figure 4b describes the positive charging into Nb:STO close to the interface to compensate the FE polarization pointing away from the substrate (P+), while a negatively charged LSMO interface stabilizes the FE state pointing toward the substrate (P−). These results are consistent with previous findings when metal/FE interfaces feature positive or negative charging in order to stabilize opposed FE states.^[^ ^25^, ^34^, ^46^, ^47^, ^57^, ^60^ ^]^

The substrate‐induced band bending at the bottom interface appears as an essential condition to stabilization of the FE state, facilitating the accumulation of *negative*/positive charge sheets at the surface/bottom interface to stabilize the *P+*/P− FE polarization.

On the other hand, the quantity Δ_BE_(C) = C 1*s* ^(UP)^ − C 1*s* ^(DW^ significantly deviates from the value of *Λ* = 1.25 eV, derived from ARPES surface sensitive measurements and expected for the band alignment at the upper interface. Its significantly smaller value Δ_BE_(C) = 0.3 eV (Figure S8, Supporting Information) indicates the band bending toward higher BEs in the DW sample and toward lower BEs in UP. Such opposite variations of V(*z*) clarifies the mechanism for FE state stabilization,^[^ ^25^ ^]^ with the charged contamination species providing the required fixed dipoles to stabilize the FE state.^[^ ^44^, ^58^, ^61^ ^]^ However, the fact that the FE‐induced band bending is lost at the surface suggests that they may simply be not enough to fully compensate for DF.

In the measurements performed at RT, presented in Figure 4d,e for Pb 4*f* respectively Ti 2*p* the same trend is identified, featured however by a smaller band offset at the PZT surface, *Λ* = 0.7 eV deduced from the ARPES measurements (Figure S9, Supporting Information), Δ_BE_ (Pb) = 0.6 eV and Δ_BE_ (Ti) = 0.3 eV. Since *W*_f_ should not depend on temperature, this implies that the decrease of *Λ* and Δ_BE_s relates to changes in the FE nature of PZT. Indeed, in Figure 4d we identify the development of an additional component at lower BEs in the Pb 4*f* spectra indicating the clustering of metallic Pb resulted from broken Pb‐O bonds,^[^ ^16^, ^44^ ^]^ accompanied by formation of oxygen vacancies (OVs) in the UP sample. The absence of such a component in the DW sample indicates that the OVs and the accompanying negative charges developing into the UP sample are a requirement to compensate for the DF and stabilize the P+ FE state. They accumulate close to the PZT surface to create a negative charge sheet which screens the fixed, polarization charges. In the DW sample featured by P− FE state on the other hand, the DF generated by the fixed negative polarization charges at the PZT surface and positive at the bottom interface with LSMO are screened by the creation of CVs (Pb and Ti).

Such ferroelectric‐dependent composition is confirmed from evaluating the stoichiometry using a monochromatized laboratory X‐ray source (Al K_*α*_ = 1486.74 eV from Kratos Analytical Ltd.). Due to its flux, orders of magnitude smaller compared to the synchrotron radiation facilities, the additional beam damage effects are negligible. Based on the data presented in Figure S10, Supporting Information, we derive the O/Pb and O/(Ti+Zr) ratios in UP and DW samples which are collected in **Table** **2**.

**Table 2:** Experimental stoichiometry. Atomic ratios derived from XPS measurements at room temperature of the UP and DW samples

	IDEAL	DW	UP
O/Pb	3.00	3.26	2.40
O/(Ti+Zr)	3.00	4.28	3.01

While the O/Ti ratio in the UP sample is 3.01, close to the ideal value of 3, in DW it is 4.28, indicating a rather large Ti and Zr deficiency. O/Pb ratio indicates the same trend, with creation of Pb vacancies in the DW sample and OVs in UP sample. Such deviations of the Pb, Ti and Zr ratio from the ideal values should impact the crystallinity of the sample through defect formation. The fact that the ARPES images from Figure 2 are sharp and well‐resolved on both samples, suggests that such defects accumulate more toward the bottom electrode, outside the thickness probed in the ARPES measurements. This means that the UP/DW samples stabilize their well‐defined FE states already during their growth at the temperature exceeding their Curie temperature T_C_,^[^ ^27^ ^]^ with the identified altered stoichiometry appearing as a fundamental requirement for screening of the DF and stabilization of the FE state.

However, we may reasonably assume that the additional generation of OVs, or CVs and their migration at RT, accompanied by alteration of the ideal stoichiometry are detrimental to the FE polarization, a fact seen in the smaller band offset, and FE‐induced band bending, Δ_BE_ (Pb) and Δ_BE_ (Ti).

Nevertheless, the fact that the measurements performed at *T* = 12K and at RT are featured by only quantitative variations of *Λ*, Δ_BE_s values is a convincing evidence that the band bending profiles keep similar shape, although with reduced curvature, indicating that the FE state of UP/DW state remains the same P+/P− both at RT and at *T* = 12 K.

On the other hand one cannot *a‐priori* exclude that the subtle balance between the positive and negative charges generated under the X‐ray beam may influence the screening of the DF by building up additional charges which may migrate and accumulate at both faces of the FE layer, eventually altering the FE phase.^[^ ^16^, ^62^ ^]^ Previous studies have also identified development of OVs accompanied by migration and clustering of metallic Pb^[^ ^16^, ^62^ ^]^ under irradiation with intense X‐ray synchrotron radiation and possible lost out of plane polarization state.

A more detailed view on the dynamics of charge creation under the X‐ray beam and of the compensation mechanisms of the FE states, extracted from the resonant photoemission performed at the *L* absorption edge of Ti is discussed in the following section. The technique offers further insight into the physics of PZT being particularly effective in separating the band‐resolved spectral signature of Ti in different valence states.^[^ ^33^, ^63^, ^64^ ^]^

### 2.3 Effect of X‐Ray Irradiation; Oxygen and Cation Vacancies

Exploring the nature of the defects developing under intense synchrotron X‐ray flux is informative in our case for at least two reasons: i) firstly, it emphasizes the mechanism of FE state stabilization ramping up as a reaction to the gradual creation of charged OVs and CVs; ii) secondly, certifies that the measurements correspond unambiguously to well‐defined FE state of PZT which do not change (remains stable) under X‐ray exposure.

**Figure 5:**
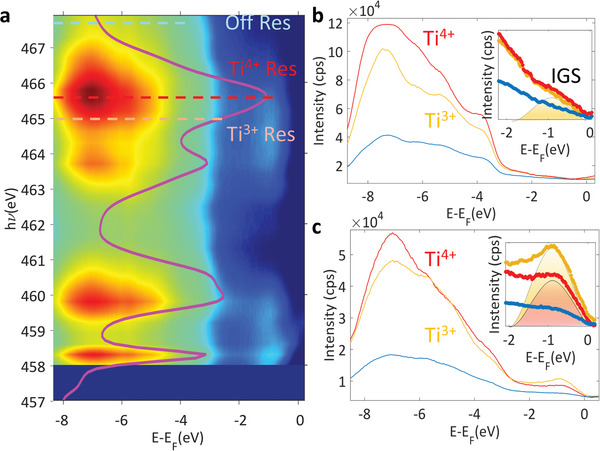
Resonant angle‐integrated intensity within the VB recorded across the Ti L_2,3_ absorption edge for UP sample (a). Overlaid is the X‐ray absorption spectrum recorded across the same energy interval. Angle integrated spectra of the VBM in resonance with Ti^3+^ and Ti^4+^ absorption (yellow and orange lines in a) and outside the resonance (blue line in a) at *T* = 12 K (b) and at room temperature (c).

**Figure** **5**a presents the angle‐integrated resonant photoemission (ResPE) intensity within the VB for UP sample, recorded in saturation conditions at RT readily identifying a strong signal at ≈0.80 eV below *E*_F_.

The ResPE spectra near the VBM recorded at the temperature 12 K (Figure 5b) and RT (Figure 5c) are collected using energies resonant with the dipole allowed 2*p*‐3*d* transitions of Ti^3+/4+^ (ℎ*ν* = 465 eV), and outside the resonance (ℎ*ν* = 467.5 eV) marked with dotted lines in Figure 5a. They show the development of in‐gap states (IGS) spectral weight signatures significantly increasing at RT.

Such in‐gap states have been observed in SrTiO_3_,^[^ ^65^ ^]^ BaTiO_3_^[^ ^66^ ^]^ and at SrTiO_3_‐based interfaces^[^ ^33^, ^63^, ^64^, ^67^ ^]^ and their origin identified in OV states created either under X‐ray irradiation or annealing.

For our UP samples, the candidates to these states may be the OVs, CVs, metallic Pb or combinations of them. We can exclude metallic Pb generated by the X‐ray exposure since the IGS signal should have been accompanied by intensity at *E*_F_ too while as seen in the ARPES images in Figure 3b,d and in the integrated ResPE data from Figure 5b,c, this is not our case. This also excludes Ti vacancies with Ti accumulating at the surface since there is no signature of Ti^0^ resonance in the ResPE spectra. This, together with the on/off enhancement when going through the Ti^3+/4+^ resonance identifies OVs at the origin of the IGS.

It is however surprising that Ti^3+^ signal is mostly absent in the XPS spectrum (Figures S10 and S11, Supporting Information). This indicates that OVs build up only in the first 1–2 unit cells at the top PZT region and their observation requires both the surface sensitive ARPES coupled with the resonant enhancement of ResPES.^[^ ^68^ ^]^

Their weaker intensity at *T* = 12K supports our scenario with OVs generation under the X‐ray beam and their migration toward the surface in order to screen the DF and stabilize the P+ FE state in the UP sample. The migration is expected to be frozen at 12 K while at RT they can easily travel toward the surface and stabilize a sheet of negative charges.

This behavior differentiates the dynamics of OVs in PZT from STO and other STO‐based heterostructures, where upon increase of temperature the OVs created by X‐rays diffuse toward the bulk.^[^ ^64^ ^]^

**Figure 6:**
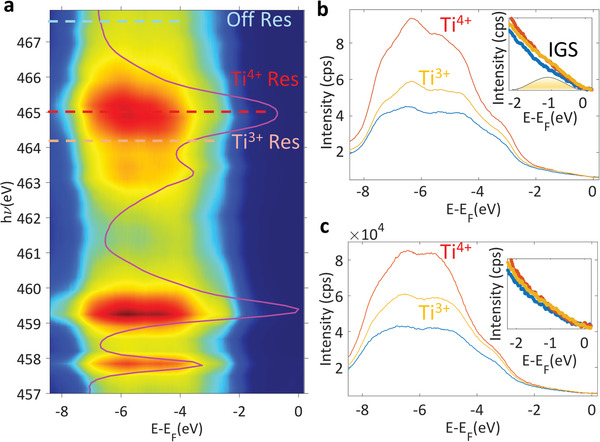
Resonant angle‐integrated intensity within the VB recorded across the Ti L_2,3_ absorption edge for DW sample (a). Overlaid is the X‐ray absorption spectrum recorded across the same energy interval. Angle integrated spectra of the VBM in resonance with Ti^3+^ and Ti^4+^ absorption (yellow and orange lines in a) and outside the resonance (blue line in a) at *T* = 12 K (b) and at room temperature (c).

In the hypothesis of OVs creation in order to stabilize the P+ FE state, no signature of IGS should be visible in the DW sample, which rather requires positive charge accumulation close to the surface. Indeed, as seen in **Figure** **6**a, which presents the angle‐integrated ResPE intensity within the VB for DW sample, recorded in saturation conditions at RT reveals no trace of IGS. Valence band spectra collected with on and off resonant *hv* values, marked with dashed lines in Figure 6a and corresponding to the Ti^3+^/Ti^4+^ absorption are given in Figure 6b for the *T* = 12K measurements and in Figure 6c for RT. Apart from the resonant enhancement by a factor of 3–4^[^ ^33^ ^]^ observed for the states located deeper into the VB, there is no indication of IGS formation, as expected for the P− PZT in the DW sample, when the FE polarization pointing toward the interface requires positive charge sheet toward the surface in order to screen the DF and stabilize the FE state.

These positive charges are detected in the FE‐dependent stoichiometry variations which stabilize the P− state as seen in Figures S11 and S12, Supporting Information, and the accompanying discussion.

**Figure 7:**
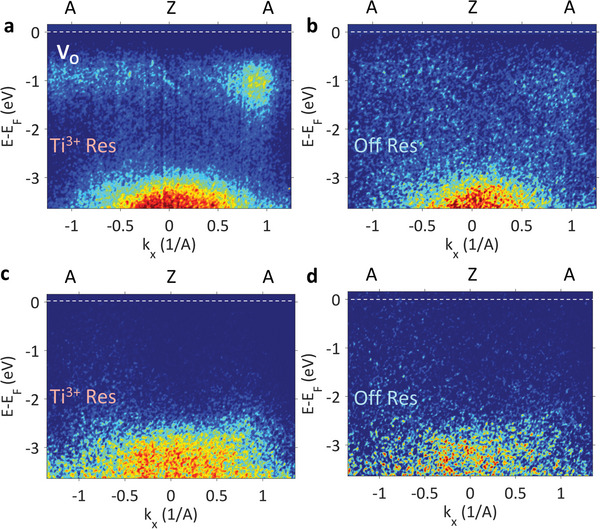
In‐gap oxygen vacancies states. ARPES images recorded at RT on UP sample using the resonant 465.0 eV energy (a) and 467.5 eV, outside the Ti L_3,2_ resonance (b). The measurements for the DW sample using the on/off resonant conditions established from the X‐ray absorption spectrum in Fig. 6a at the energies marked with dotted red (on‐resonance) and yellow (off‐resonance) lines are given respectively in (c) and (d).

The non‐dispersive character of the IGS associated with OVs can be seen in the ARPES images presented in **Figure** **7**a,b for UP and in Figure 7(c,d) for DW. The k‐resolved VB region recorded at RT along the AZA direction of the BZ identifies the non‐dispersive OV‐related band, manifesting the standard flat signature of a defect state.^[^ ^31^, ^33^, ^69^, ^70^ ^]^

We may compare the formation of such impurity bands in our P+ PZT with the extensively studied case of STO. There, similar to PZT, the OV site may be either occupied by two electrons or may have single occupancy with the additional electron released into an itinerant 2D electron system (2DES). The onset between localization of the doubly occupied OV with filled Ti 3d *e_g_* orbitals and itinerancy of *t_2g_* electrons corresponding to singly occupied OV is given by the increase of the electronic correlations.^[^ ^71^, ^72^ ^]^ Moreover, it has been shown that the energy of the IGS in STO depends on the strength of the electronic correlations, moving it away from *E*_F_ as the correlations strength increases.^[^ ^70^, ^73^, ^74^ ^]^ Compared to STO, where the position of the IGS is reported at ≈1.3 eV below *E*_F_,^[^ ^75^ ^]^ in our case, the IGS of PZT at ≈0.85 eV as seen in in Figure 5 and in Figure 7a,b suggests weaker correlation effects in PZT compared to STO.

Indeed, from Figures 5 and 7 it is evident the absence of any itinerant *t_2g_*‐derived bands in the form of 2D electron system (2DES) since there is no intensity at the *E*_F_. With the presence of IGS only having *e_g_* character it is reasonable to assume that PZT is a weakly correlated system, and the electronic correlations are not strong enough to drive the system into a dichotomic regime of localized *e_g_* states away from *E*_F_ and itinerant electrons into *t_2g_* orbitals at *E*_F_ like in STO^[^ ^65^, ^69^, ^75^ ^]^ or STO‐based interfaces.^[^ ^33^, ^76^ ^]^ In addition, we expect that the IGS identified in PZT to carry significant magnetic moments, in the range of 0.1–0.3 µ_B_ similar to the STO case.^[^ ^70^, ^72^, ^74^ ^]^

The observed dynamic effects of gradual negative carriers generation in the form of OVs under the X‐ray beam as a requirement to stabilize and maintain the FE state is characteristic to FE PZT, However, we emphasize that this gradually developing mechanism can be extended to other perturbations to which the FE system must adapt, for example, to the applied bias which switches the FE polarization between opposed states, which should be accompanied by dynamic creation of positive or negative charges which migrate through the bulk and redistribute close to the surfaces.

## 3 Outlook and Conclusions

Our pioneering SX‐ARPES measurements on strained thin films of PZT grown on STO and LSMO substrates establish the intrinsic k‐resolved electronic structure of this paradigm FE material in two opposite polarization states. The large photoelectron escape depth achieved in the soft‐X‐ray energy range has been essential for resolve the 3D band structure as well as reconstruct the electrostatic potential profile across the PZT films and their band offset to the substrates. Moreover, resonant ARPES at the Ti *L*‐edge has elucidated the Ti character of the delocalized and localized defect valence states.

These results have informed, first, about the mobile and fixed charges involved in the stabilization of the FE polarization and in screening of the DF. We have found that the negative charges near the surface, required to stabilize the P+ state of PZT grown on n‐doped Nb:STO, are *V*_O_s. In the ARPES data, they are identified as non‐dispersive Ti‐derived localized states falling into the PZT band gap at 0.8 eV below *E*_F_. The PZT film accumulates this surface charge by diffusion of *V*_O_s toward the surface, as evidenced by the dynamics of their resonance response in the Ti *L*‐edge spectra under X‐ray irradiation. In analogy to STO, we conjecture that the *V*_O_s in PZT may form ferromagnetic clusters. In turn, the positive charges required to stabilize the P− state of PZT grown on LSMO, are generated by a self‐doping mechanism through creation of Pb, Ti and Zr vacancies as derived from our quantitative XPS measurements.

The second aspect of our findings is the interaction of PZT with the substrate, crucial for the stabilization of either P+ or P− polarization state. From the experimental k‐resolved band structure, we find that the crystallographic structure of the substrate propagates through the full thickness of our PZT thin films. Specifically, the PZT films inherit the TG structure when grown on Nb:STO, and the RH structure when grown on LSMO as manifested by replica bands in the ARPES spectra. Such a structural shift goes beyond the conventional treatments of this material assuming only its small deviations from the pseudocubic structure.

In a wide perspective of our findings, the observed effect of doping with anion and cation vacancies induced by the FE polarization can be utilized for tuning electronic structure of ferroelectric and multiferroic layers in heterostructures. Such a way of defect engineering may find applications, for example, in energy storage and conversion materials where creating localized electron energy levels is a way to enhance absorption of light in the visible range. Furthermore, the energy levels of *V*_O_s, if in ferromagnetic clusters and properly aligned in energy, can be used for spin injection in FE heterostructures. The observed structural variance of FE materials, in turn, can find applications in spintronics. For example, they can be interfaced to non‐collinear spin antiferromagnets, where symmetry‐controlled propagation of the FE instability across the interface can modify the period of the spin cycloid^[^ ^77^, ^78^, ^79^ ^]^ or generate topologically non‐trivial spin textures.^[^ ^80^ ^]^ For the RH‐distorted polar perovskites, this can be achieved by selecting one of the four FE displacements along the [111] diagonals of the pseudocubic unit cell, while the TG structures display distortions along the *c*‐axis only. Furthermore, the incorporation of atoms with high atomic number (Z) into the FE materials possessing inherent breaking of the inversion symmetry can constitute a route to enhance spin–orbit interaction effects where the spin–split bands carry locked spins and even net spin currents.

## 4 Experimental Section

### Pulsed Laser Deposition

Single crystalline STO(001) and Nb:STO (001) substrates with a miscut angle of 0.05–0.2° (CrysTec, Berlin) were used to prepare the investigated structures. In order to obtain high quality thin films on single crystalline STO and Nb:STO substrates, preliminary substrate preparations were performed. They consist in the transformation of the optically polished surface into a stepped and terraced surface, which is well ordered at the atomic scale. For this purpose, STO substrates were etched in NH_4_‐HF solution for 15 s in order to remove Sr residues and then thermally treated for ≈4 h at elevated 1000 °C. In this manner a purely TiO_2_‐terminated surface was obtained. All resulting steps are approximately equal in height (single unit cell ≈ 0.4 nm), parallel, and equidistant.

Atomic force microscopy images of etched substrates before the PLD deposition and of the resulting PZT layers are given in Figure S13, Supporting Information.

Ablation of commercial PZT and LSMO targets from Praxair was performed using a KrF laser (*λ* = 248 nm) with a repetition rate of 1 Hz for LSMO and 5 Hz for PZT at a laser fluence of 2 J cm^−2^. During LSMO deposition, the substrate was kept at *T* = 700 °C and then decreased with a ramp of 10 °C min^−1^ to 575 °C for the PZT layer. On the Nb:STO substrate PZT was grown at *T* = 575 °C. The oxygen pressure during the deposition was 0.2 mbar for PZT and 0.27 mbar for LSMO. After deposition, PZT films were post‐annealed in the deposition chamber at 575 °C for 1 h in 1 bar O_2_ atmosphere. They were then transferred in the N_2_ atmosphere from the preparation chamber to the analysis chamber, which was observed to bring only minimal surface contamination, easily overcome by the large probing depth of the soft X‐ray range.

### X‐Ray Diffraction

X‐ray diffraction studies (XRD) were performed at room temperature on a Rigaku SmartLab diffractometer in high resolution settings (X‐ray mirror and two bounce Ge(220) monochromator, l_K*α*1_ = 1.5406 Å). The reciprocal space mapping (RSM) was performed on a Bruker D8 Advance diffractometer (Bruker AXS GmbH Germany) with copper anode X‐ray tube in medium resolution parallel beam setting (X‐ray mirror and nickel filter; l_K*α*1_ = 1.5406 Å, l_K*α*2_ = 1.5445 Å, l_Kb_ = 1.3922 Å).

### Density Functional Theory

The calculations were performed within the generalized gradient approximation (GGA) using the quantum ESPRESSO plane‐wave code,^[^ ^79^ ^]^ and the exchange and correlations functional in the Perdew–Burke–Ernzerhof (PBE) parametrization. Norm conserving pseudopotentials from PseusoDojo were used.^[^ ^80^ ^]^

Ti—Zr substitutional doping was treated by means of virtual crystal approximation (VCA) replacing each A‐site of the perovskite with a fictitious atom with fractional valence, instead of explicit doping which would require a significant computational burden due to larger supercells.

The kinetic‐energy cut‐off for the plane waves was set at 60 Ry and for the charge density at 240 Ry. The BZ integration was performed on an automatically generated Monkhorst–Pack 10 × 10 × 10 *k*‐mesh, with Gaussian energy level smearing of 0.02 Ry.

Then, the internal coordinates were relaxed at the experimental *c*/*a* = 1.07 ratio, with the in‐plane parameters resulting from the cell relaxation. Visualization of the crystalline structures had been performed by using the Vesta software.^[^ ^83^ ^]^

### ARPES Measurements

SX‐ARPES experiments were carried out at ADRESS beamline at Swiss Light Source which delivers high photon flux in soft X‐ray range,^[^ ^81^ ^]^ allowing the band structure investigation of 3D materials with the additional benefit of a remarkably sharp momentum resolution along the out‐of‐plane direction *k*_z_ and thus full 3D momentum.^[^ ^82^ ^]^ The relationship between electron momentum (*k*_||_,*k*_⊥_), photoelectron kinetic energy, and the photoelectron emission angle are given in Ref. [81]. ARPES measurements had been performed in pressure better than 10^−11^ mbar, at the temperature of *T* = 12 K and at *T* = 300 K. Fermi level was calibrated using a silver foil in electrical contact with the sample. The combined resolution (thermal broadening in addition to the photon beam and the ARPES analyzer) was of ≈70 meV.

The XAS measurements had been performed in total electron yield mode, by measuring the secondary electrons current photogenerated in the sample, with the angle between the sample and incident X‐rays of 23°. X‐ray linear dichroism (XLD) signal, which is the footprint of orbital energy and occupation, is calculated as the difference between the normalized signal generated by linear horizontal and linear vertical‐polarized radiation on the sample.

## Conflict of Interest

The authors declare no conflict of interest.

## Supporting information

Supporting information

## Data Availability

The data that support the findings of this study are available from the corresponding author upon reasonable request.
