# Supplementary material for: Experimental Band Structure of Pb(Zr,Ti)O3: Mechanism of Ferroelectric Stabilization
Source: Adv Sci (Weinh). 2023 Jan 2;10(6):2205476. doi: 10.1002/advs.202205476 (PMC9951575; doi:10.1002/advs.202205476)
Supplement: Supplementary file 1 — Supporting information [file ADVS-10-2205476-s001.pdf]

Supporting Information

1. X-ray diffraction

The symmetric 2θ-ω XRD scans results included in Supplementary Fig. 1a show a good crystalline similarity of both PZT films, with slightly broader peaks of DW ($FWHM_{S1}$=2.543^o) than UP ($FWHM_{S2}$=2.308^o) due to possibly larger internal microstrains[1] in DW. The RSM in the vicinity of the -103 node of STO (Supplementary Fig.1b) and Nb:STO (Supplementary Fig. 1c) shows that the PZT films in both DW and UP are fully strained at the in-plane lattice constant of the cubic STO and Nb:STO substrates. In both samples PZT feature elongated growth along the c axis, with the ratio between the out-of-plane and the in-plane lattice parameters *c/a* = 1.081 for DW and *c/a* = 1.073 for UP. This ratio exceeds the bulk value of ~1.04 - 1.05 [2,3] and enforces the stabilization of the FE phase through large off-centering of the Pb and Ti/Zr cations in the unit cell.

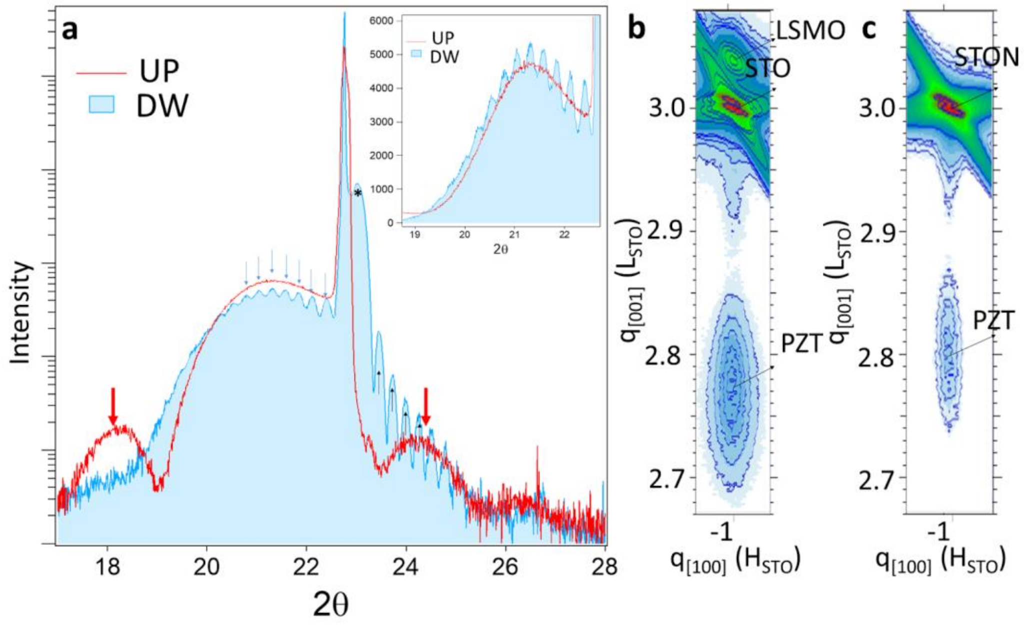

***Supplementary Figure 1. X-ray diffraction**. 2θ-ω scan for PZT grown in UP and DW samples (**a**). In the inset, the zoomed region of the 001 peak of PZT in linear scale of intensity is given for both samples. Reciprocal space mapping (RSM) scans of DW and UP samples are presented in (**b**) and respectively (**c**)*

2. Piezoresponse force microscopy

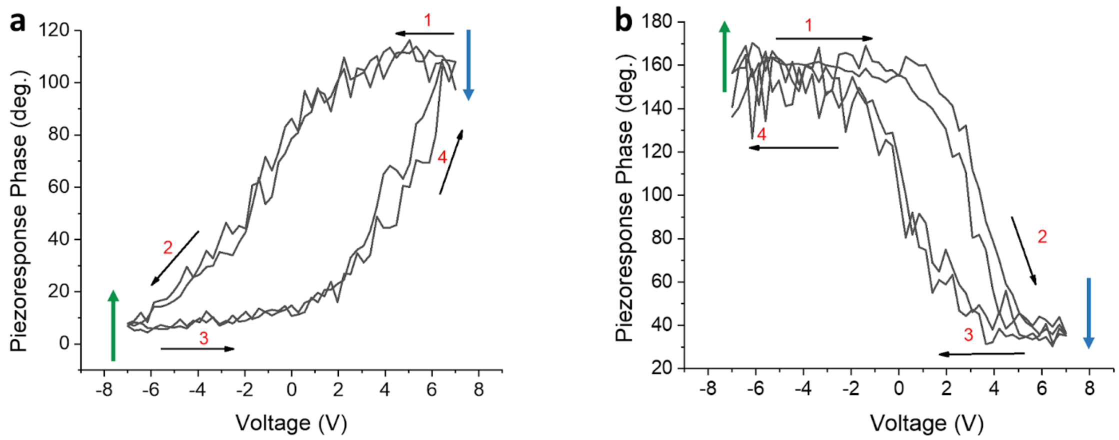

***Supplementary Figure 2 Piezoresponse phase loops** versus applied DC voltage, measured with an AC driving voltage Vac = 1.5 V for DW (**a**) and UP (**b**) samples*

Supplementary Figure 2 shows the piezoresponse phase loops acquired with a piezo force microscope for DW and UP samples as a function of the applied DC voltage. The DC voltage applied on the cantilever was swept between -7 V and +7 V starting from positive values for PZT in DW and negative values for PZT in UP as indicated by the numbered arrows.

3. iso-E surfaces of strained and relaxed PZT

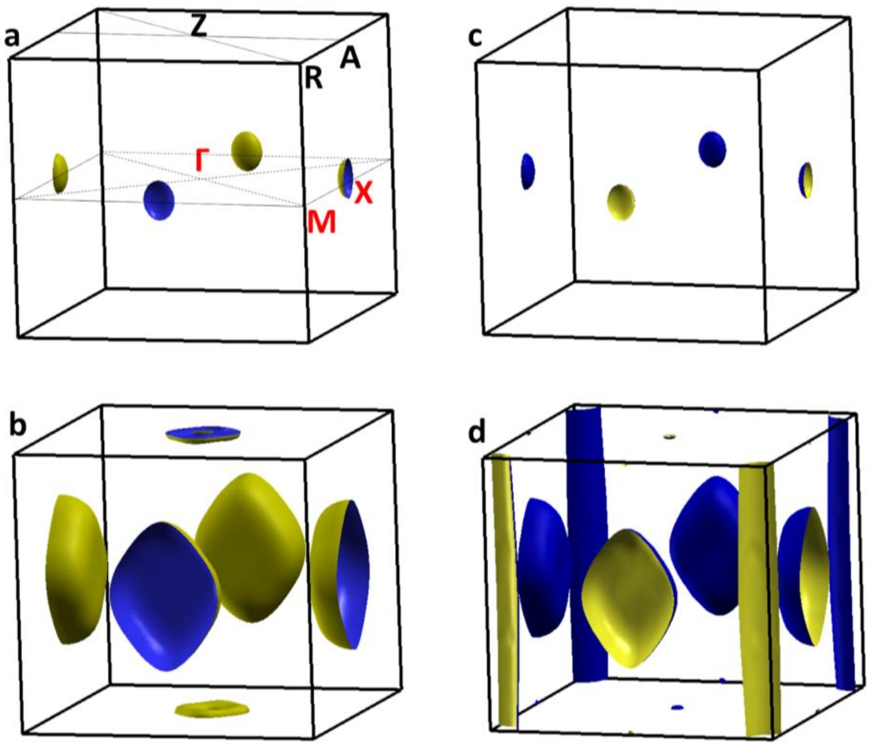

Supplementary Figure 3. Theoretical iso-E surfaces *of Pb(Zr,Ti)O_3 at (**a**) the VBM and (**b**) 0.5 eV below the VBM for the substrate - strained growth. The fully relaxed unit cell at the VMB is in (**c**) and 0.5 eV below the VBM in (**d**)*

4. Rhombohedral distortions stable at the room temperature

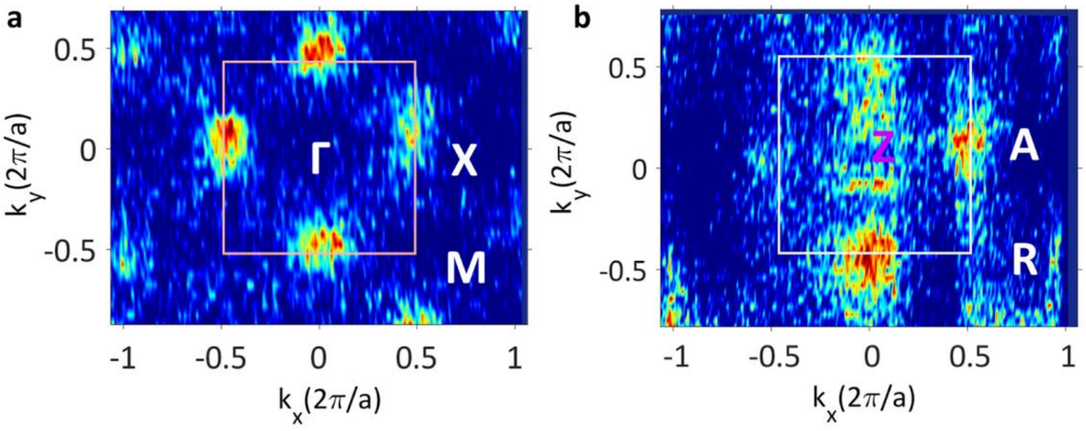

Supplementary Figure 4. Crystalline structure at room temperature. *In-plane (k_x,k_y) iso-E maps of PZT in DW recorded with h□ = 520 eV in the MΓX plane at VBM and at 0.5 eV below the VBM in the ZAR plane of the TG cell.*

5. X-ray absorption spectroscopy and linear dichroism

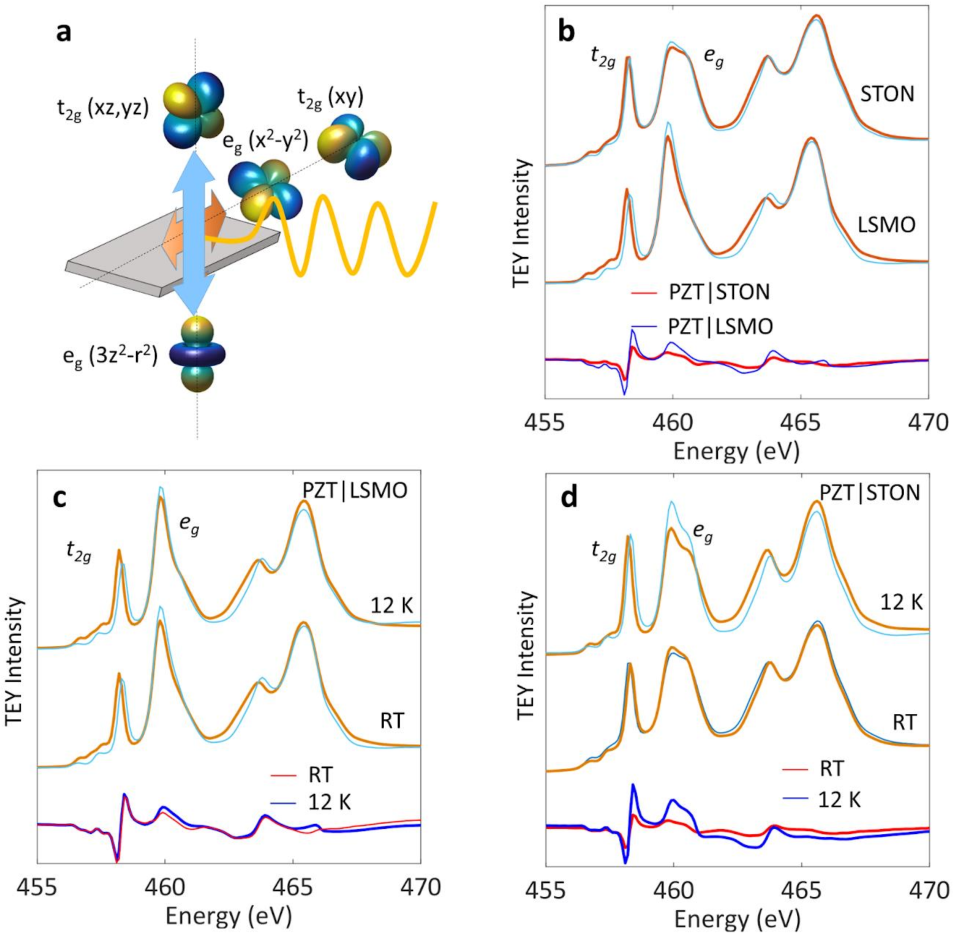

***Supplementary Figure 5. XAS spectra**. Sketch of the experimental geometry of the XAS experiment (**a**). XAS spectra recorded at room temperature for DW and UP samples (**b**). Room temperature and T=12 K XAS spectra recorded on DW (**c**) and UP (**d**) samples*

X-ray absorption (XAS) measurements at the Ti $L_{2,3}$-edge were performed at both room temperature and at T=12 K for the UP and DW samples in the geometry sketched in Supplementary Fig. 5**a**. The linearly horizontal (photon polarization parallel (*E*||*c*) with the crystal *c*-axis) and vertical (photon polarization perpendicular (*E*||*c*) with the crystal *c*-axis) radiation was used to probe the energy of orbitals with the corresponding symmetry.

Although it was hypothesized that the linear dichroism signal - the difference between signal recorded with different light polarization, recorded on FE samples may be a fingerprint of the polarization state (pointing either in plane or out of plane - inwards or outwards)[4,5], we do not identify major qualitative differences between the XAS signal recorded on our samples with opposed FE states. We find that the specific signatures and their variation with the temperature are rather a signature of the different substrate-induced distortions.

In the ideal, centrosymmetric form, the t_{2g} orbitals are degenerate. Breaking of the symmetry lifts this degeneracy such that some of the orbitals will have lower energy which should increase their occupancy. The increase or decrease of the orbital energy is seen in the XAS spectra as corresponding shifts towards higher or lower energies[6]. In our case, according to existing computations, the XLD signal is compatible with a splitting of the t_{2g} such that the d_{xy} orbitals lie ~120 meV[6] lower than d_{xz} and d_{yz}[4] for PZT in both DW and UP. Since PZT in DW and UP are in opposed FE states, their striking similarity (Supplementary Fig. 5**b**) suggest that the origin of the dichroism signal stems most likely from the different crystalline state of PZT, RH in DW and TG in UP. This is further supported by the previously established sensitivity of the TG cell at temperature variation, while the cell parameters of PZT in RH structure stayed rather insensitive to temperature changes. More precisely, it was estimated that the bulk tetragonality ratio increases from $c/a \sim 1.07$ at room temperature to ~ 1.085 close to 12 K[7] whereas the *c* parameter of the pseudocubic RH cell was not found to vary much upon cooling or heating[8]. Indeed, it is seen in Supplementary Fig. 5**c** that there is practically no difference between the XLD spectra recorded on DW at T=12K or at RT, while PZT in UP features the increase of the XLD signal by almost a factor of two (Supplementary Fig. 5**d**), as expected for higher TG distortion. Hence, this variation supports our hypothesis that it originates in a different crystalline structure rather than a different FE state.

6. Signature of the rhombohedral distortion in the band structure of PZT

The signature of RH-distorted PZT in DW, understood as a translation of the fundamental iso-*E* surface with an umklapp vector (π/a, π/a, π/a). Such translation defines a mixture of domains with three equivalent *RΓR* diagonals of the BZ[39]. In particular, such reconstruction translates the *XΓX* and *AZA* directions of the TG cell into respectively *ARA* and *XMX* ones. Their signatures are shown by white arrows in Supplementary Fig. 6 (**a**,**c**) which represent the experimental band dispersions recorded along *AZA* and *RAR* directions of the BZ for both DW and UP samples. The calculated bands are overlaid on the ARPES images: red lines correspond to the main probed direction computed for the TG cell and yellow dots correspond to the translated ones along (π/a, π/a, π/a) direction of the BZ, resulting from the RH distortion. For DW, the calculated *RAR* bandstructure does not reproduce the maximum identified experimentally in the *A* point and requires, additionally, the *ΓXΓ* projection (Supplementary Fig. 6**a**). The translation of the X point onto the A point is identified as well in the ARPES images recorded along the *AZA* direction of the TG cell at T=12 K (Supplementary Fig. 6 (**c**)) and at room temperature (Supplementary Fig. 4), which identifies the RH distortion through the translated band structure calculated along *XMX* into the *AZA* direction.

The electronic band structure of PZT in UP on the other hand is in good agreement with the band structure calculated for the TG cell, and does not present the hallmarks of the RH distortions nor along *RAR* (Supplementary Fig. 6**b**) or *AZA* directions (Supplementary Fig. 6**d**) indicating that PZT in UP sample is in the TG phase.

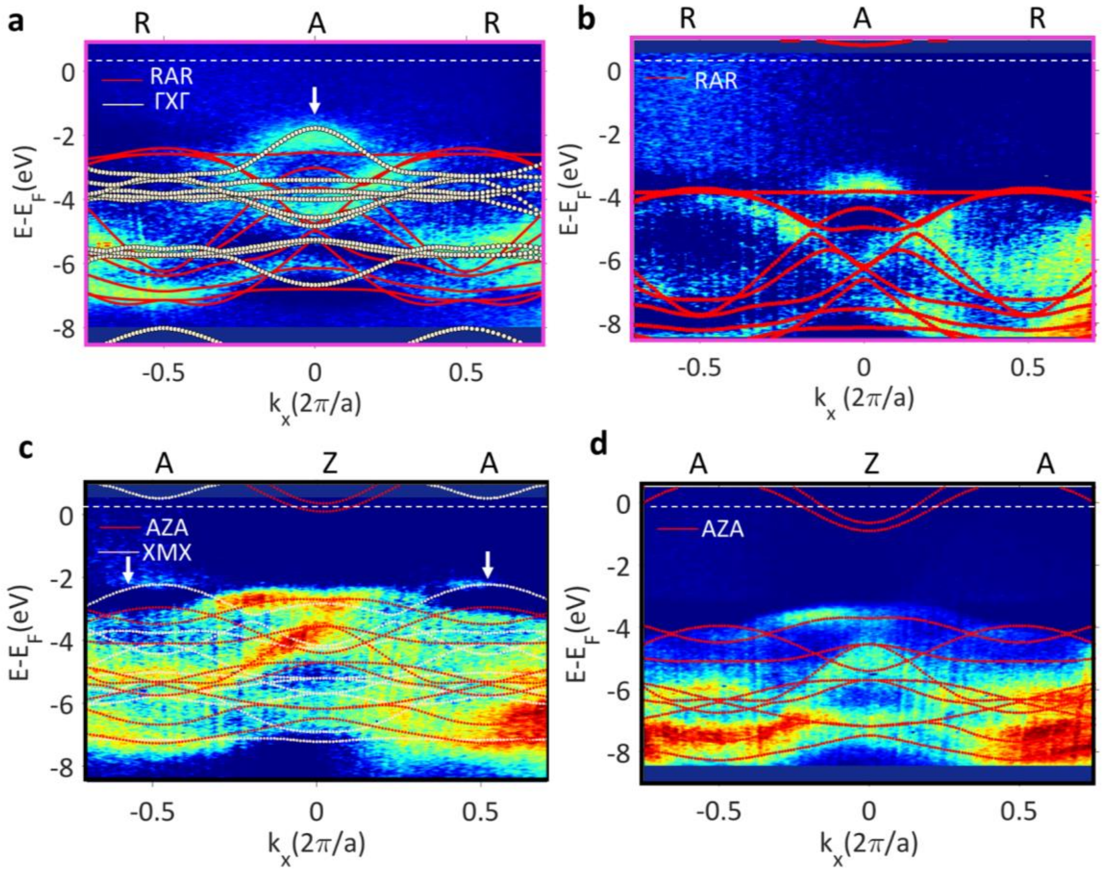

Supplementary Figure 6. Experimental band structure *of PZT recorded along RAR (**a,b**) and AZA (**c,d**) directions of the TG-cell BZ at the temperature T=12K for DW (**a**,**c**) and UP (**b**,**d**) samples. The calculated band structures are overlaid with red lines for the TG aristotype directions and yellow circles for the RH-reconstructed unit cell*

7. **k**-resolved electronic density of states

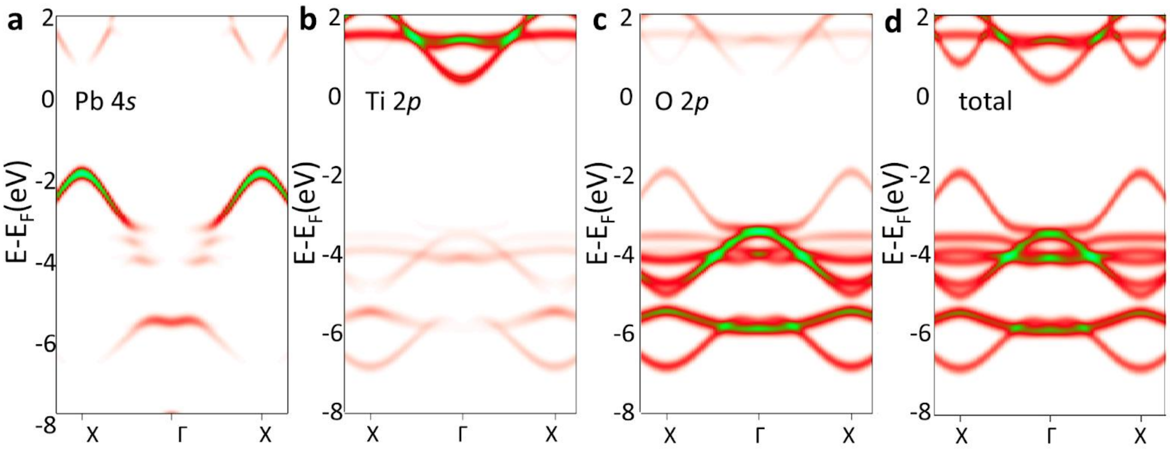

***Supplementary Figure 7**. **Theoretical electronic structure** of ferroelectric Pb(Zr,Ti)O_3 along the XΓX direction. **k**-resolved projected density of states for Pb 4s (**a**), Ti 2p (b), O 2p (c) and total density of states (**d**)*

8. Time dependence of contamination

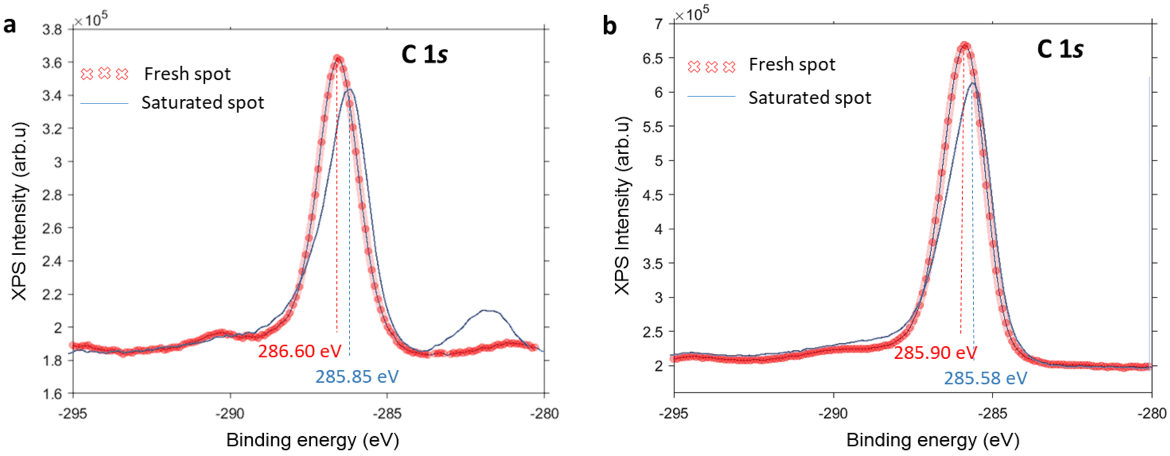

***Supplementary Figure 8. C1s XPS spectra** recorded on UP (**a**) and DW (**b**) samples in a fresh spot and at saturation, after 20 mins. of continuous exposure to the X-ray beam*

9. Band structure recorded at the room temperature

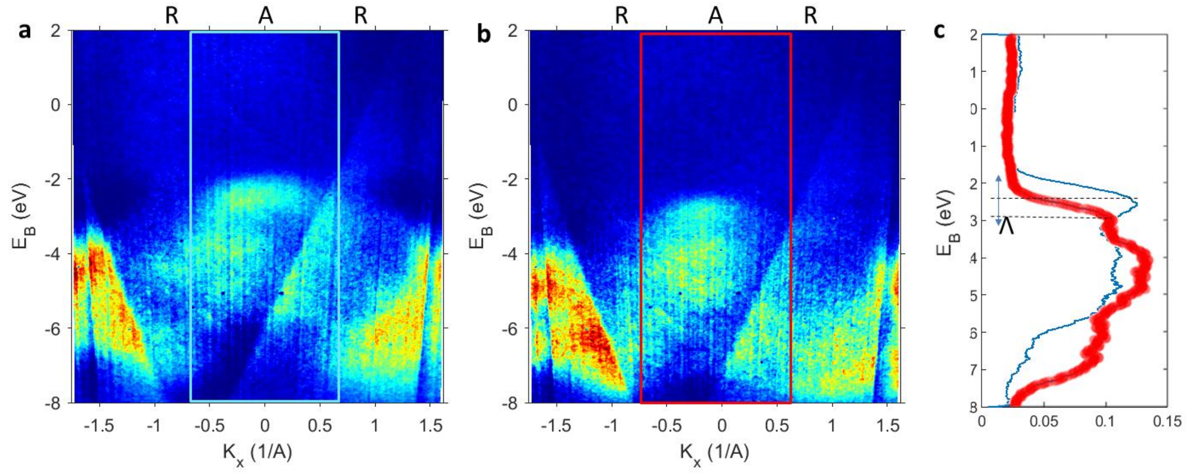

Supplementary Figure 9. ARPES images recorded at RT on UP (a) and DW (b) samples. Energy distribution curves obtained by integrating the ARPES signal in the **k**-region marked with blue and red are given in (**c**)

10. Experimental stoichiometry from XPS measurements

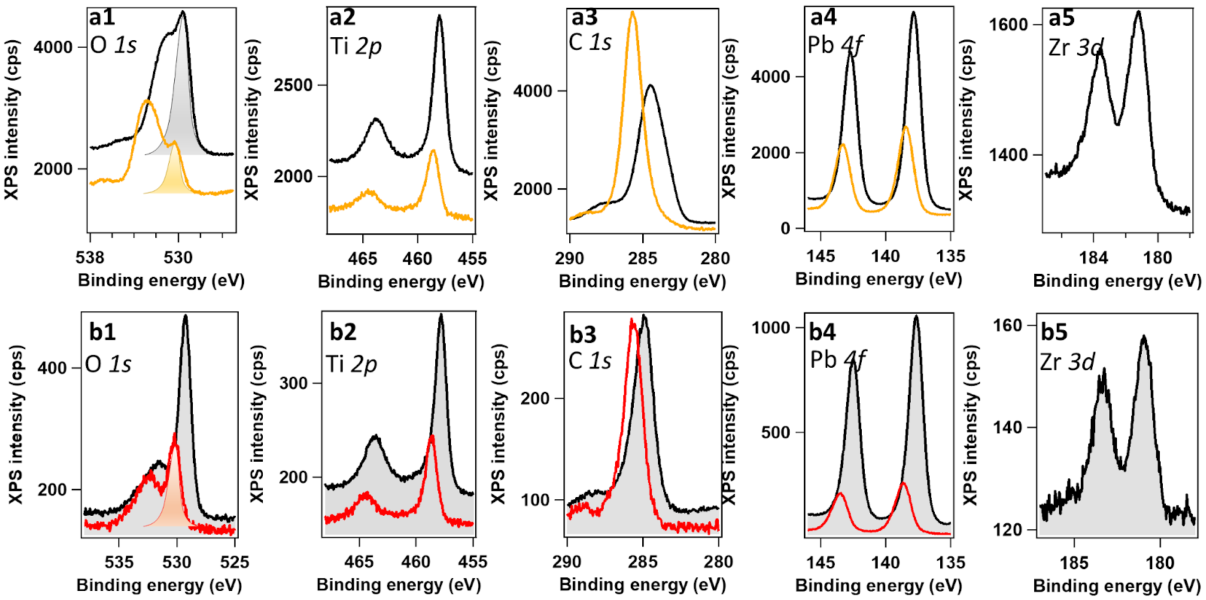

***Supplementary Figure 10. Surface and bulk-sensitive laboratory XPS spectra** recorded in bulk (black lines) and surface sensitive (orange/red) geometry for P+ PZT on STON (a1-a5) and P- PZT on LSMO (b1-b5)*

	on LSMO; P-	on Nb:STO; P+
	surface	surface
O/Pb	2.60	1.50
O/(Ti+Zr)	3.55	2.75

Supplementary Table 1. O/Pb and O/(Ti+Zr) atomic ratios from surface sensitive measurements

Surface-sensitive XPS measurements, performed by tilting the sample at 60° with respect to the analyser plane indicate strong oxygen deficient character in UP sample and Pb deficiency in the DW sample

11. Time evolution of stoichiometry from synchrotron radiation measurements

The dynamics of defect creation is deduced from the variation of the Pb 4*f* and Ti 2*p* spectra in the UP and DW samples recorded on a spot freshly exposed to X-ray beam and at saturation, after 20 mins. of continuous exposure. We define the relative variation of the peak integral amplitude as $\varepsilon_A=(A^{sat.}-A^{fresh})/A^{fresh}$, where A^{sat} is the area recorded at saturation and A^{fresh} is the corresponding peak area evaluated in a fresh spot, during the first 60s. of exposure. The Ti 2*p*, and Pb 4*f* spectra and their decomposition in components are presented in Supplementary Fig. 11 with C 1*s* given in Supplementary Fig. 8. From Supplementary Table2, which gives the relative variations of their integral amplitudes before and after exposure we identify the increase of both Pb 4*f* and Ti 2*p* signal, accompanied by the decrease of C 1*s* intensity in the DW sample.

	$\varepsilon_A^{Ti\ 2p}$ (%)	$\varepsilon_A^{Pb\ 4f}$ (%)	$\varepsilon_A^{C\ 1s}$ (%)
**DW**	+3.9	+4.5	-7.8%

**UP**	–12.9	-6.3	-9.1%

Supplementary Table 2. Integral areas. Relative variation of integral areas derived from XPS measurements at room temperature of the UP and DW samples before and after X-ray irradiation.

Such a variation is expected assuming that under the X-ray beam, the C-C and C-O bonds of the contamination layer break, leaving the sample. From the intensity variations of Pb 4*f* and C 1*s* peaks recorded in a fresh spot, we estimate a thickness of the contamination layer d_C^{DW} ~5.66 nm[9], decreasing with approximately 2% during the irradiation, at saturation. The corresponding attenuation due to the substrate layer reduces and the 4%-5% larger amplitudes of both Pb 4*f* and Ti 2*p* peaks are consistent with the thinner contamination layer. The same trend is observed in the evolution of C 1*s* area of the UP sample, where the estimated initial thickness d_C^{UP}~3.75 nm decreases to d_C^{UP}~3.68 nm under the X-ray beam. On the other hand, the evolution of Ti 2*p* and Pb 4*f* spectra, by decreasing with 6.3 % (Pb) and 12.3% (Ti) indicate the evolution into significant cation deficiency at saturation. We exclude the beam damage effect as a cause of such variation, since similar trends should have been observed in both UP and DW samples. The most plausible explanation is that under the X-ray beam, concomitant CVs and OVs develop in the UP sample, and migrate towards the surface (OVs) and bottom interface (CVs) in order to screen the additional charges created during the photoemission process (secondary electrons and photo-holes). This mechanism actively prevents the loss of the FE polarization while maintaining the same P+ FE state derived from the initial substrate-induced band bending.

On the other hand, it seems that in the DW sample the initial off-stoichiometry identified from laboratory XPS measurements, with slight CV accumulation, and nominal positive charge at the surface is enough to compensate for the additional carrier dynamics induced by the continuous exposure to the synchrotron ray beam. Hence, creation of additional charged species in the form of OVs or CVs is not required.

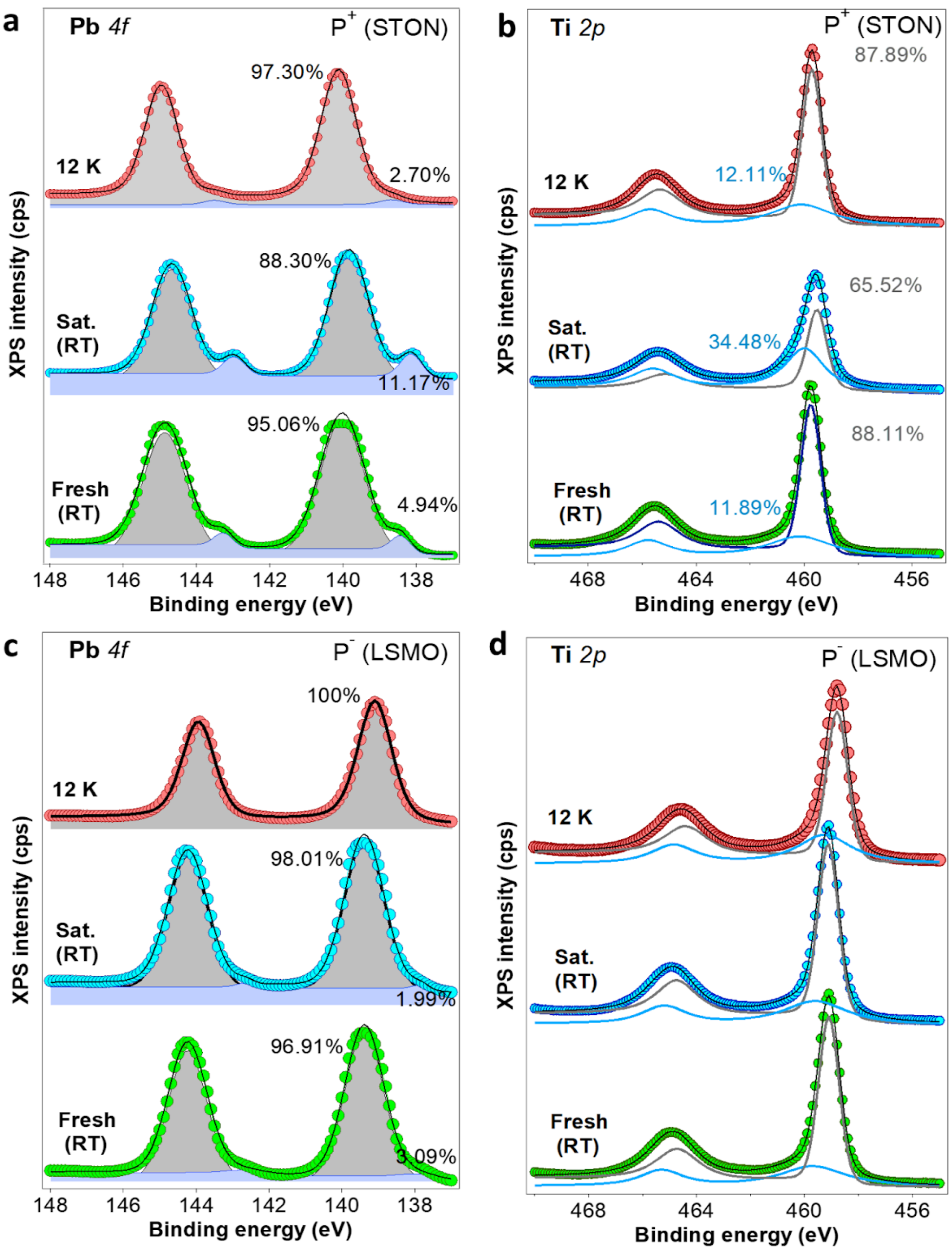

Supplementary Figure 11. XPS spectra *of Pb 4f (a,c) and Ti 2p (b,d) core levels recorded on UP sample (**a**,**b**) and on DW (**c**,**d**). The experimental data are simulated with Voigt lines*

12. Time evolution of P+/P- states from synchrotron radiation measurements

Indeed, the irradiation-time evolution of the Ti *2p* spectra in DW sample (Supplementary Fig. 12**a**) recorded in a spot freshly exposed to the X-ray beam show remarkable stability during X-ray exposure. These spectra were recorded at each 60 s and are featured by a main component at lower binding energies (LBE) associated to Ti^{4+} states at 459 eV, accompanied by a smaller component at 0.85 eV higher binding energies (HBE), gradually developing during irradiation. However, the HBE amplitude accounts for at most 2% of the total amplitude, saturating after the first 300 s of measurements (Fig. 4c). We assign the signals of the LBE and HBE components to regions with out-of-plane FE polarization oriented away from the surface (P-) and towards the surface (P+) respectively, causing upwards or downwards band bending [16,25]. The HBE component at approximately 1 eV away from the main line is also consistent with the separation in BE between the signals coming from regions with P+ and P- [10-11].

While the BE evolution for the LBE and HBE peaks (Supplementary Fig. 12**b**) reflects the change of the FE-induced band bending at the surface of PZT, the evolution of their integral intensity (Supplementary Fig. 12**c**) describes the size evolution of the regions with different FE polarization, showing a gradual inflation of the P+ regions to ~2% of the total polarized volume under X-ray exposure. Hence, our XPS analysis supports a scenario where initially the PZT film in DW sample is stabilized mostly in a P- state due to the adsorption of polar molecules at its surface and screening charges from the bottom LSMO electrode. The secondary electrons and the holes generated in the photoemission process gradually compensate for the DF in a fraction of 2% P+ regions. The opposite **P** projection on the z axis in these regions gives competing contributions to the final band bending [17].

The development of P+ regions immersed into the P- states for the DW sample is schematically shown in Supplementary Fig. 12**d**.

For the UP sample on the other hand, the irradiation-time evolution of the Ti 2*p* core levels (Supplementary Fig. 11) shows that at the P+ surface the HBE component develops, saturating at much higher intensity. Accounting for 34% of the total Ti 2*p* intensity, this indicates formation of large regions with overcompensated DF (P++), developing in response to the charge generated by the X-ray beam as sketched in Supplementary Fig. 12**e**. Such a picture is consistent with previous findings on thin FE films, where, although formation of FE domains is prevented, vortex-like FE patterns may develop[17] and they are featured by the FE polarization gradually rotating going from the bottom electrode towards the surface of the film.

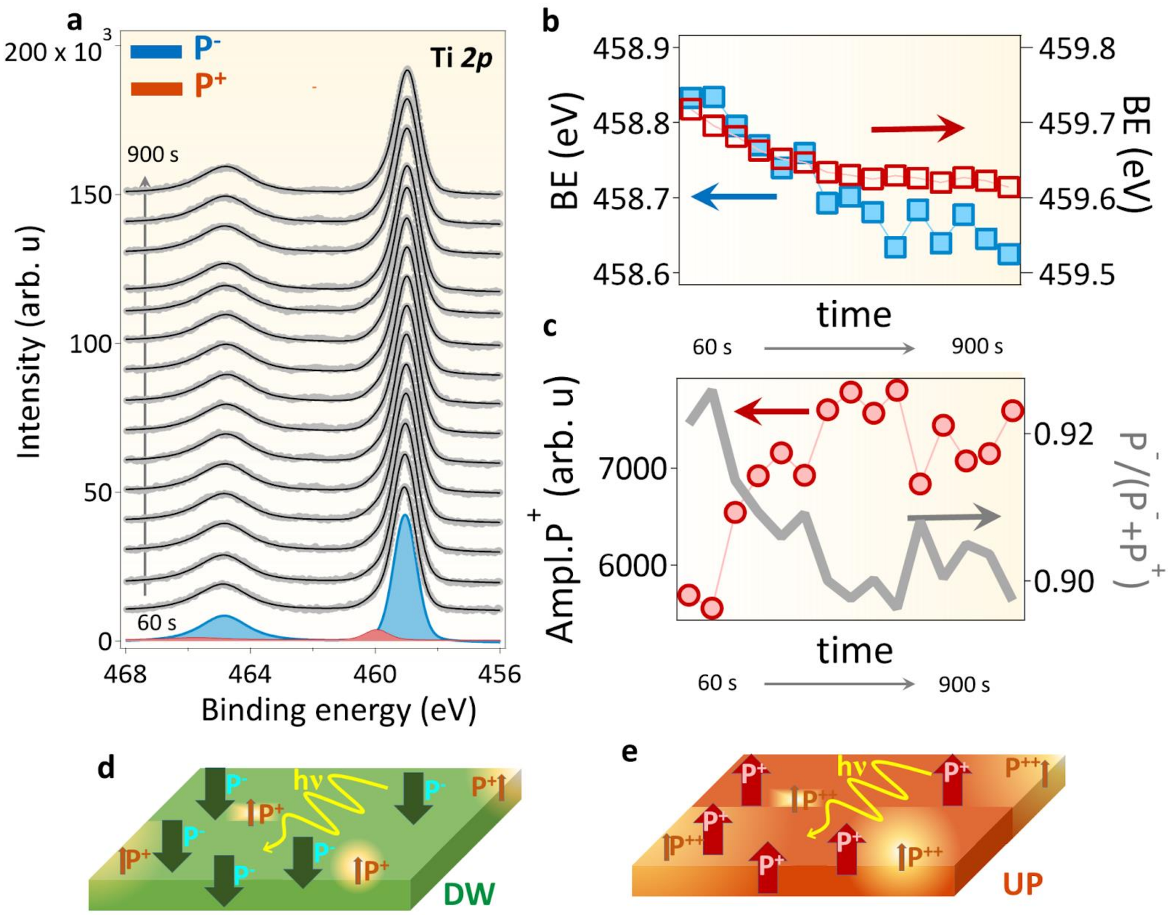

***Supplementary Figure 12 X-ray exposure dependence**. Time evolution of Ti 2p core level of the DW sample (**a**). Time evolution of the BE for both high- and low-BE components (**b**) and of their corresponding integral amplitudes (**c**). Sketch of the coexisting P- and P+ FE phases for DW (**d**) and of coexisting P+ and overcompensated P++ regions in UP (**e**)*

13. Morphology of the substrate and of the PZT films

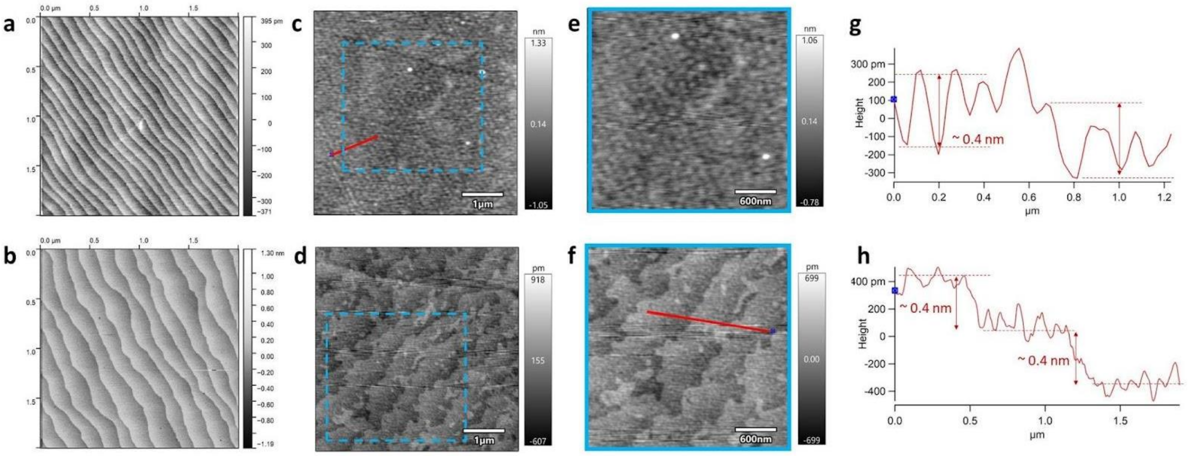

Supplementary Figure 13. Surface morphology *Atomic force microscopy - AFM images of the etched STO (**a**) and Nb:STO (**b**) before the PLD deposition. AFM images on PZT in DW (**c**) and UP (**d**) samples and zoom of the regions marked with blue square in respectively (**e**) and (**f**). Line profiles recorded along the red lines are presented in (**g**) for PZT in DW and (**h**) for PZT in the UP sample.*

Supplementary References

[1] I. Pasuk, F. Neațu, Ștefan Neațu, M. Florea, C. M. Istrate, I. Pintilie, L. Pintilie, *Nanomaterials (Basel)* **2021**, *11*.

[2] I. Kanno, H. Kotera, K. Wasa, T. Matsunaga, T. Kamada, R. Takayama, *J. Appl. Phys.* **2003**, *93*, 4091.

[3] V. Nagarajan, J. Junquera, J. Q. He, C. L. Jia, R. Waser, K. Lee, Y. K. Kim, S. Baik, T. Zhao, R. Ramesh, P. Ghosez, K. M. Rabe, *J. Appl. Phys.* **2006**, *100*, 051609.

[4] E. Arenholz, G. Van der Laan, A. Fraile-Rodríguez, P. Yu, Q. He, R. Ramesh, *Phys. Rev. B: Condens. Matter Mater. Phys.* **2010**, *82*, 140103.

[5] S. Polisetty, J. Zhou, J. Karthik, A. R. Damodaran, D. Chen, A. Scholl, L. W. Martin, M. Holcomb, *J. Phys. Condens. Matter* **2012**, *24*, 245902.

[6] M. Wu, H. L. Xin, J. O. Wang, X. J. Li, X. B. Yuan, H. Zeng, J. C. Zheng, H. Q. Wang, *J. Synchrotron Radiat.* **2018**, *25*, 777.

[7] T. Nishimatsu, K. Aoyagi, T. Kiguchi, T. J. Konno, Y. Kawazoe, H. Funakubo, A. Kumar, U. V. Waghmare, *J. Phys. Soc. Jpn.* **2012**, *81*, 124702.

[8] A. Vigliante, U. Gebhardt, A. Rühm, P. Wochner, F. S. Razavi, H. U. Habermeier, *EPL* **2001**, *54*, 619.
[9] A. Jablonski, J. Zemek, *Surf. Interface Anal.* **2009**, *41*, 193.
[10] E. Kröger, A. Petraru, A. Quer, R. Soni, M. Kalläne, N. A. Pertsev, H. Kohlstedt, K. Rossnagel, *Phys. Rev. B Condens. Matter* **2016**, *93*, 235415.
[11] J. E. Rault, G. Agnus, T. Maroutian, V. Pillard, P. Lecoeur, G. Niu, B. Vilquin, M. G. Silly, A. Bendounan, F. Sirotti, N. Barrett, *Phys. Rev. B Condens. Matter* **2013**, *87*, 155146.
